# Injection augmentation and endoscopic repair of type 1 laryngeal clefts: development of a management algorithm

**DOI:** 10.1186/s40463-020-00447-0

**Published:** 2020-07-14

**Authors:** Andre Isaac, Orysya Svystun, Wendy Johannsen, Hamdy El-Hakim

**Affiliations:** 1 Pediatric Otolaryngology, Division of Otolaryngology Head & Neck Surgery, Departments of Surgery & Pediatrics, The Stollery Children’s Hospital, University of Alberta, Edmonton, Alberta Canada; 2 Faculty of Medicine & Dentistry, University of Alberta, Edmonton, Alberta Canada; 3 Outpatient Feeding & Swallowing Service, The Stollery Children’s Hospital, University of Alberta, Edmonton, Alberta Canada

**Keywords:** Dysphagia, Laryngeal cleft, Swallowing disorders, Pediatrics

## Abstract

**Objectives:**

To describe indications for injection augmentation (IA), endoscopic repair (ER) and conservative methods for the management of type 1 laryngeal cleft (LC1) and propose a management algorithm. We also aimed to compare success of IA and ER and determine independent predictors of treatment failure.

**Methods:**

Retrospective study of patients diagnosed with LC1 at a Pediatric Otolaryngology referral centre between 2004 and 2016. All had pre-operative instrumental swallowing evaluation (VFSS/FEES), and were managed with a combination of conservative measures, IA and/or ER. We collected demographics, symptoms, comorbidities, VFSS/FEES results, and operative details. The primary outcome was symptom resolution by parental report. The secondary outcome was predictors of treatment failure.

**Results:**

88 patients were included in the analysis, with mean age 26 ± 25 months. Most presented with choking events (68%) or recurrent pneumonias (48%). In total, there were 55 IA performed and 45 ER. Of the patients who received IA, 19 required subsequent ER. 95% had symptom improvement, 67% had complete resolution. IA had a 56% long-term success rate, whereas that for ER was 85%. Tube feeding at initial evaluation was an independent predictor of treatment failure (HR 11.33 [1.51–84.97], *p* = 0.018).

**Conclusions:**

LC1 can be effectively managed with a combination of IA and ER with favorable results. Failure to respond to IA does not preclude ER, and both have their role in management. Patients who are tube fed have a higher probability of treatment failure. We propose a management algorithm that includes reasoning for conservative approaches, and reduces exposure to general anesthesia.

## Introduction

Swallowing dysfunction (SD) is a common problem in children and its causes can be complex and multifactorial. Laryngeal cleft (LC) is an important anomaly that can be associated with SD and has gained increased attention in the recent literature. The most widely accepted classification system for LC was described by Benjamin and Inglis in 1989 [1], and includes four types: type 1 LC (LC1) being a dehiscence in the inter-arytenoid muscle resulting in a low inter-arytenoid notch; type 2 (LC2) involving the cricoid cartilage; type 3 (LC3) extending into the cervical trachea; and type 4 (LC4) involving the intra-thoracic trachea and possibly the carina. LC1 in particular poses a diagnostic challenge [2, 3]. It has been linked with clinical problems such as recurrent aspiration pneumonia, choking during feeding, stridor, and failure to thrive. Given that in the last decade there has been a surge in the literature on LC1 with an increase in diagnosis and management, some authors believe this has been a previously under-diagnosed and under-treated problem [4]. Whilst several authors have advocated their own diagnostic and therapeutic strategies, a recent consensus guideline from the International Pediatric Otolaryngology working group found a wide variation in practice among experts [5].

Management strategies for LC1 have so far included non-surgical methods such as thickened or modified feeds, alternate route to oral feeding, as well as surgical options including open or endoscopic repair (ER) [6, 7]. More recently, injection augmentation (IA) (also known as injection laryngoplasty) has emerged as an alternative surgical strategy for LC1 with several authors reporting favourable outcomes [8–12]. However, currently there is no consensus in the literature as to the optimal management of LC1 patients in terms of threshold and timing of using various medical and surgical strategies. There is particular controversy around IA, with some authors advocating its use as a diagnostic and/or prognostic technique [10], whereas others use it as a purely therapeutic procedure, or a combination of the above [9]. The majority of the literature did not directly compare IA with ER, nor did they have strict criteria of when or why either procedure should be pursued. In addition, few authors provided details on how long conservative measures should be attempted before proceeding with surgery, the role of a single step diagnostic and therapeutic endoscopic examination, and decision-making around repeat injections or repairs for patients whose symptoms recur. There is also variation in practice when it comes to special populations such as syndromic or neurologically impaired patients, as most studies on the topic included a heterogeneous mix of medically complex patients with comorbidities including neurological impairment [9, 10, 12, 13]. Finally, little is known about the parameters of patients who respond to or fail treatment and whether this can be predicted based on pre-operative variables.

The objective of this study was to describe the indications of IA, ER and conservative methods for the management of LC1 through a retrospective review of medically and surgically treated patients, and the creation of a management algorithm based on our experience. We also aimed to directly compare the long-term success of IA and ER in achieving symptom resolution, as well as determine independent predictors of failure to respond to treatment.

## Methods

This was a retrospective case series, based on reviewing a prospectively collected clinical and surgical database at a tertiary Pediatric Otolaryngology referral centre. The eligible patients were those children who were evaluated at the multidisciplinary swallowing clinic at the Stollery Children’s Hospital between January and December 2016. We only included children who presented with symptoms suggestive of SD, underwent pre-operative instrumental swallowing evaluation (videofluoroscopic swallowing study (VFSS) and/ or functional endoscopic evaluation of swallowing (FEES)) and had a diagnostic suspension laryngoscopy and bronchoscopy (SLB) where palpation of the inter-arytenoid area suggested the diagnosis of LC1. Swallowing evaluation was performed and interpreted by a certified pediatric Speech and Language Pathologist (SLP) in conjunction with a pediatric radiologist, or a Pediatric Otolaryngologist as per established protocols [14]. Patients for which a baseline VFSS/FEES was unavailable, those with previous airway surgery, and patients with less than three months of follow-up were excluded.

We collected the parameters and variables from the surgical database, electronic medical records and private practice records. These included demographic information, presenting symptoms (history of choking and cough upon feeding and swallowing, stridor, chest infections or pneumonia, apparent life threatening episodes, cyanotic spells), prematurity at birth (< 36 week gestation), gastroesophageal reflux disease- GERD (based on clinical symptoms, response to proton pump inhibitors), asthma and/ or atopy (based on clinical history and response to bronchodilators, family history), failure to thrive (<5th WHO percentile for weight or crossing down more than two percentiles consecutively), obesity (>95th WHO percentile for age and sex), admission to intensive care unit with or without intubation, history of named neurologic impairment, syndromes or dysmorphism, developmental delay (failure to progress to several mile stones) and prior or concomitant surgery. Preoperative use of an alternate route of feeding (for example nasogastric or gastrostomy tube), as opposed to oral unmodified or modified intake was recorded. VFSS/FEES data collected included presence of residues, penetration and/or aspiration (silent or overt). The type of procedure(s) performed was recorded, as well as the response to treatment and its effect on the route of feeding or feeds modification.

**Fig. 1:**
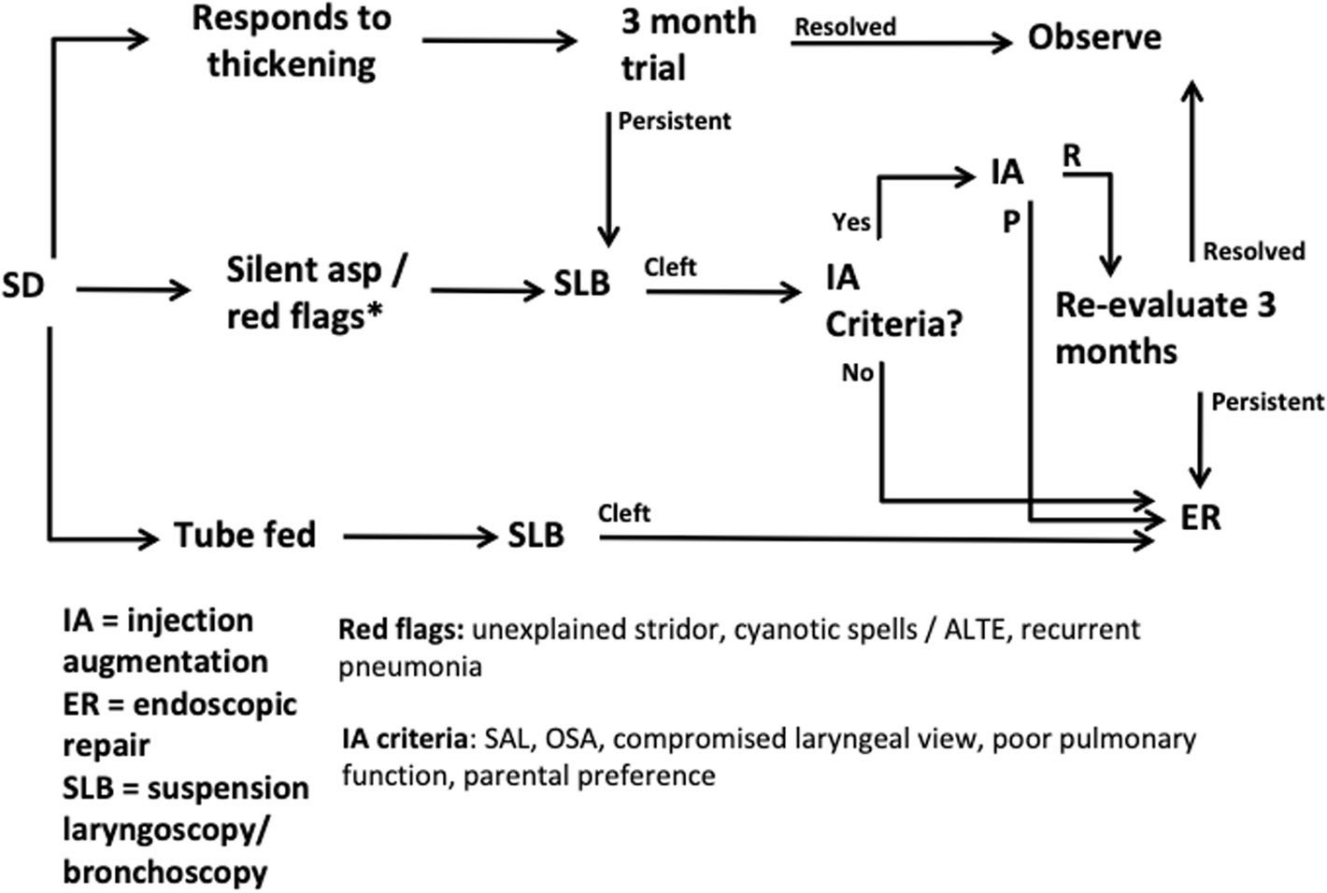
Management algorithm for LC1 patients. SD = swallowing dysfunction; SLB = suspension laryngoscopy and bronchoscopy; IA = injection augmentation; ER = endoscopic repair

The general outline of management followed in our multi-disciplinary clinic was described by Syystun et al. [15]. Largely, where possible a trial of oral thickened liquid intake was undertaken for a period of three months until resolution of symptoms was achieved. Following that an attempt to wean the patient gradually was directed by SLP. The use of tube feeding is restricted to those patients who silently aspirated on all consistencies on instrumental testing, or presented with ALTE’s, cyanotic spells, or FTT. This is further delineated in our management algorithm which is shown in Fig. 1. Of note, the decision to take a patient to the operating room for SLB is based on many factors, including results of instrumental swallowing evaluation as well as clinical history and physical examination. This is especially important for patients with only mildly abnormal swallowing examinations but severe clinical symptoms or history such as witnessed blue spells, ALTE’s, frequent recurrent pneumonias, etc.

**Fig. 2:**
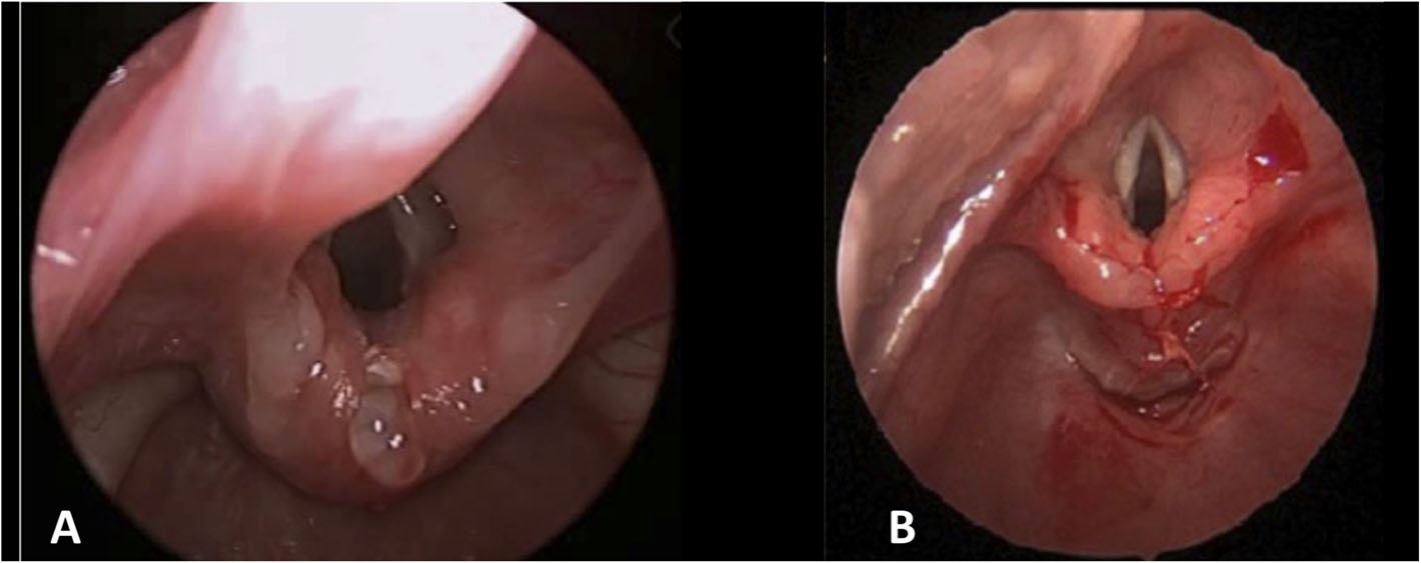
Post-operative photo of LC1 after injection augmentation (**a**), and after endoscopic repair (**b**)

IA was performed endoscopically with a 22 g butterfly needle using a hyaluronic acid-based filler (Deflux: Red Leaf Medical, Mississauga, ON). The volume injected varied from 0.2 to 0.4 ml. The exact volume injected was based on the surgeon’s judgement. The aim was to augment the posterior inter-arytenoid space to well above the level of the vocal cords without causing swelling of the tissue anteriorly into the airway (Fig. 2a). ER was performed using a cold steel technique and 7.0 polydioxanone sutures. A vocal cord spreader was used to splay the arytenoids, then basket forceps and endoscopic scissors were used to create a linear incision in the inter-arytenoid area (coronal plane). The tissue was then brought together in the sagittal plane using inverted simple sutures from deep to superficial, usually four sutures per patient (Fig. 2b).

We proposed ER for all patients who failed conservative measures with feeding modification, patients who were tube fed at initial evaluation, and for patients who failed or relapsed after IA. Meanwhile, we performed IA in cases where ER would have proved technically difficult or unsafe, such as patients with an anterior larynx or compromised laryngeal view, those with secondary airway lesions, and those with poor pulmonary health or respiratory reserve causing difficulty in maintaining a tubeless field, uninterruptedly while breathing spontaneously for long enough to perform a repair. For some patients this decision was made prior to surgery, and for some this was an intra-operative decision. We also used IA pragmatically, as part of a diagnostic/therapeutic SLB when a LC1 was identified but an ER was not planned. These were usually patients for which a LC1 was not suspected based on pre-operative FEES/VFSS nonetheless it was definitively identified intra-operatively. As such it can be difficult both from an operating room time/duration perspective as well as a consent process to plan and perform ER in these instances. Finally, parental preference of IA vs ER was also considered in the final decisions.

The *main outcome* measure was parental reporting on symptom resolution and/or improvement. Complete resolution was defined as achieving full oral feeding with no coughing/choking on all consistencies, no requirement of feeds modification, and no recurrence of baseline symptoms (choking, coughing, cyanosis, ALTE, recurrent chest infections). Symptom improvement was defined as any significant reduction in baseline symptoms AND no recurrent pneumonias or dangerous/red flag signs or symptoms. Failure was defined as persistent dependence on tube feeding or the same regiment of modified oral feeding and or persistent clinical symptoms. Since this was a retrospectively designed study, post-operative VFSS/FEES data was not always available. At our centre, we aim to limit the use of VFSS due to concerns of radiation exposure from repeated examinations. Post-operative FEES data was available for some patients but not all, for several reasons. We did not routinely perform FEES post-operatively unless the pre-operative evaluation showed silent aspiration or aspiration with only subtle symptoms, or if there was unreliable/inconsistent reporting by parents. In other instances we performed a bedside clinical swallowing evaluation with SLP to determine safety for oral feeding, and only performed FEES if indicated. FEES clinic appointments also require more resources and time than a regular post-operative visit, thus resource allocation sometimes played into the decision to perform FEES after treatment. Finally, we also employ a shared decision-making model with the parents when performing post-operative examinations. As such, many parents prefered to forego FEES if their child is clinically improved and passes a bedside swallowing evaluation. Unless the clinician had a clinical reason to warrant a FEES (ex: silent aspiration on pre-operative FEES/VFSS), a shared decision between the parent and clinician was undertaken. Overall, post-operative instrumental swallowing evaluation was done pragmatically; thus, post-operative VFSS/FEES variables were not an essential end point for the purposes of this retrospective study. However where post-operative VFSS/FEES data were available, they were collected and analyzed as a separate group. The *secondary outcome measure* was identifying the independent predictors of failure of ER or IA.

Basic descriptive statistics were used to analyze the parameters of the patient cohort, the overall outcomes, and the success of IA and ER. Given the skewed distribution of the sample, non-parametric descriptive statistics were used. We then performed a binary logistic regression to identify independent predictors of treatment failure. All variables with *p* < 0.1 correlation on univariate analysis as well as any clinically relevant variables were included in the regression. The variables that met these criteria and were included in the regression included patient age, presence of silent aspiration, recurrent pneumonias, alternate route to oral feeding, and presence of ALTEs. All analyses were carried out using SPSS Version 23.

### Ethical considerations

The project was approved by the local Research Ethics Board (Pro00070412). As this was a non-interventional, retrospective observational study, the only risk to participants was related to patient confidentiality and privacy. As such, all data was anonymized after extraction, and kept in encrypted files on password-protected hard drives as per local ethical review board standards. All study personnel were trained on local health privacy protection and information laws and guidelines.

#### Results

Of the approximately 1000 new patients seen in the aspiration clinic during the study time period, one hundred and one patients with LC1 were identified and reviewed. Of these, eight were excluded due to lack of follow-up and five were excluded due to lack of preoperative VFSS/FEES data. Eighty-eight patients met all criteria and were included in the analysis. Demographic information and past medical history as well as secondary airway lesions are available in Table 1. The median age was 18 months (range 2–99 months), with 53% males. The median follow-up time was 14 months (range 4–54 months, IQR 12 months). The majority of patients (68%) presented with a history of choking on liquids or solids (Table 1). Nearly one half of the patients (48%) presented with recurrent pneumonias. Eleven patients (13%) presented with cyanotic spells and 3 (3%) had ALTEs. In terms of comorbidities, the most common were GERD (53%) and SDB (25%). Eight percent had neurological impairment and 10% were dysmorphic or syndromic. Secondary airway lesions were uncommon and included tracheomalacia (6%) and subglottic stenosis (3%), see Table 1. On initial VFSS/FEES, the most common finding was laryngeal penetration (88%, Table 1). There was also a high rate of silent aspiration (39%). Thirteen patients had an alternate route to oral feeding at initial evaluation.

**Table 1:** Demographic, past medical history, and instrumental swallowing assessment (FEES or VFSS) information for patient cohort

Parameters		N of 88 (%)
**Median age (range)**		18 months (2–99)
**Males**		54 (61%)
**Median follow-up (range)**		14 months (4–54)
**Comorbidity**	SDB	22 (25%)
	Asthma	4 (5%)
	GERD	47 (53%)
	Neurological impairment	7 (8%)
	Dysmorphism/syndrome	9 (10%)
	Ex-prematurity	21 (24%)
	None	19 (22%)
**Secondary airway lesion**	Laryngomalacia	2 (2%)
	Laryngeal dyskinesia/immobility	3 (3%)
	Vocal cord nodules	2 (2%)
	TEF	2 (2%)
	Tracheomalacia	5 6%)
**Presenting symptom**	Choking (liquids)	60 (68%)
	Choking (solids)	33 (38%)
	Recurrent pneumonia	42 (48%)
	Stridor	28 (32%)
	Cyanotic spells	11 (13%)
	ALTE	3 (3%)
**Instrumental swallowing assessment findings**	Laryngeal penetration	77 (88%)
	Aspiration	37 (42%)
	Silent aspiration	34 (39%)

**Fig. 3:**
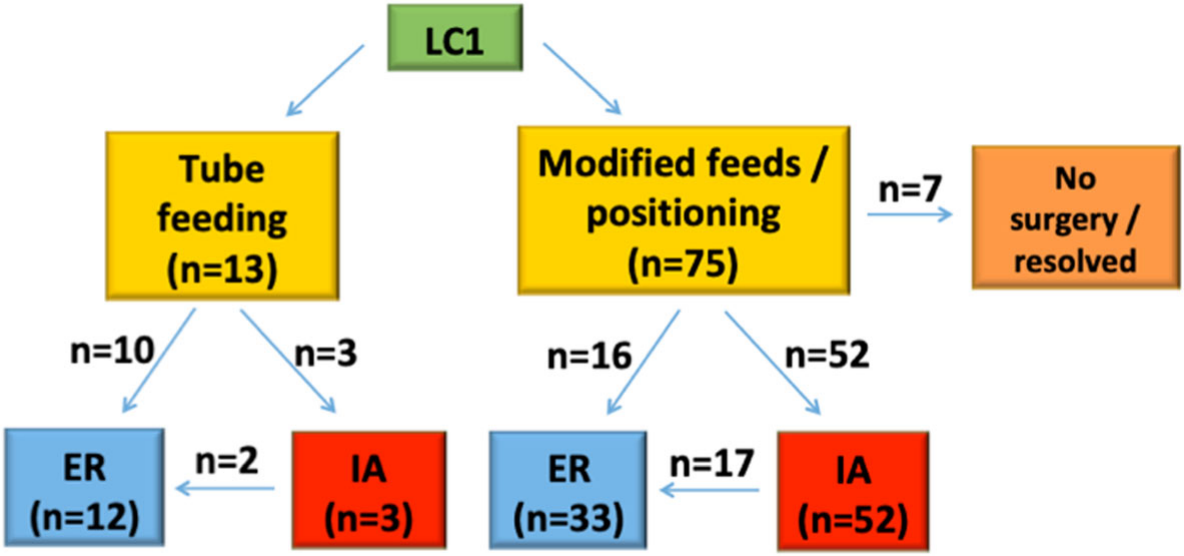
Summary of management of tube-fed and non-tube fed LC1 patients. LC1 = type 1 laryngeal cleft; IA = injection augmentation; ER = endoscopic repair

**Fig. 4:**
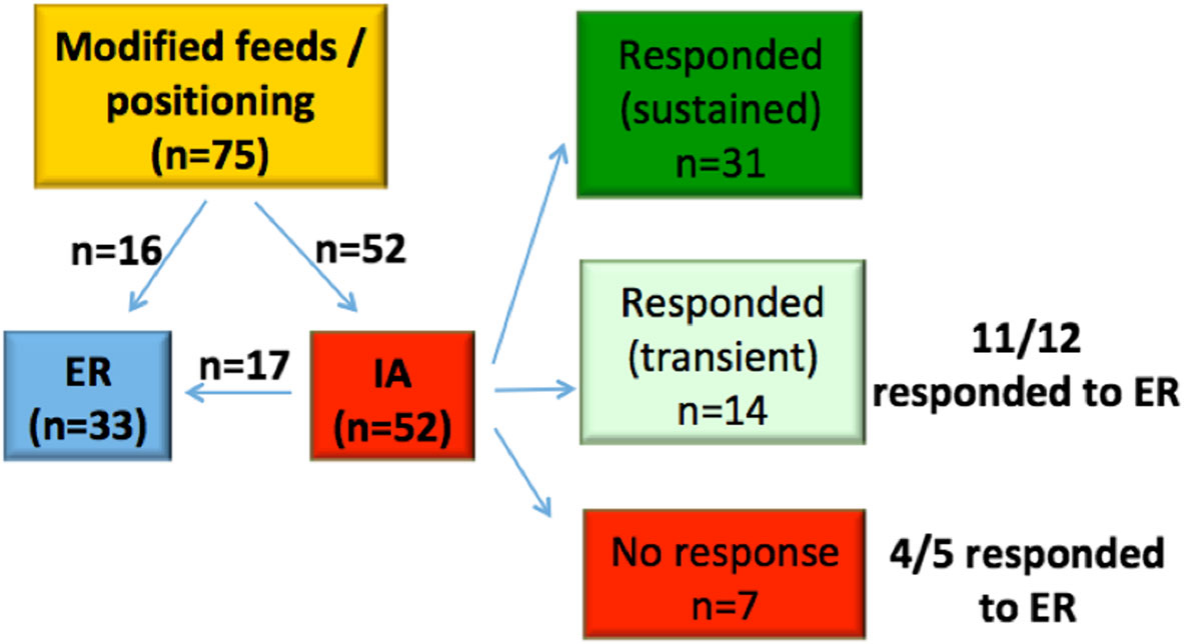
Summary of non-tube fed LC1 patients managed initially with IA. LC1 = type 1 laryngeal cleft; ER = endoscopic repair; IA = injection augmentation

A summary of how patients were managed is presented in Figs. 3 and 4. In total, there were 55 IA performed, and 45 ER. Of the patients who received IA, 19 relapsed and required ER at a later date. A summary of how the IA patients were managed in terms of response and progression to ER is shown in Fig. 4. Notably, of the seven patients who did not respond favourably to IA, five had ER and four of these responded well with symptoms resolution. Not all patients who failed (or only partially responded) to IA went on to have ER. This was for a variety of reasons including medical fitness, technical difficulty in accessing the larynx to perform ER, as well as patient/parental preference.

The details of response to treatment are shown in Table 2. Overall, 95% of patients had at least symptom improvement, 67% had complete symptom resolution, and 98% were feeding orally at least partially. Of the tube fed infants, 69% were tube-free after treatment. When IA and ER were compared directly, IA had a 56% long-term success rate in achieving complete symptom resolution, whereas ER had an 85% long-term success rate.

**Table 2:** Outcomes of entire patient cohort, and sub-divided into tube-fed and non tube-fed patients

Outcome	All Patients ***N*** = 88 (%)	Non-tube fed ***N*** = 75 (%)	Tube fed ***N*** = 13 (%)	***P***-value
Symptom improvement	84 (95%)	72 (96%)	12 (92%)	0.479
Symptom resolution	59 (67%)	52 (69%)	7 (54%)	0.341
Oral feeding	86 (98%)	75 (100%)	11 (85%)	0.020
Modified feeds	16 (18%)	12 (16%)	4 (31%)	0.243
G/NG-tube introduced	4 (5%)	4 (5%)	N/A	N/A
G/NG-tube removed	9 (10%)	N/A	9 (69%)	N/A

Post-operative instrumental swallowing evaluation was available for 27 patients. The results of these are presented in Table 3, along with a comparison with the pre-operative findings for this patient cohort. Of these, 14 had improved swallow studies, with 10 of the patients having a normal swallow postoperatively.

**Table 3:** Post-operative instrumental swallowing evaluation results

Clinical Finding	Pre-operative (***N*** = 27)	Post-operative (***N*** = 27)	***P***-value
Normal	0 (0%)	10 (37%)	0.001
Laryngeal penetration	11 (41%)	12 (44%)	1.000
Aspiration	16 (59%)	5 (19%)	0.005

The results of the multivariate binary logistic regression are shown in Table 4. The only independent predictor of failure of treatment was tube feeding at initial evaluation (hazard ratio 11.33 [1.51–84.97], *p* = 0.018).

**Table 4:** Multivariate binary logistic regression for independent predictors of failure to respond to treatment

Variable	B-coefficient [95% CI]	***P***-value
Silent aspiration	7.91 [0.75–83.47]	0.086
Alternate route	11.33 [1.51–84.97]	0.018
ALTE	31.57 [0.83–1203.12]	0.063
Recurrent pneumonia	9.32 [0.81–107.76]	0.074

Complications were uncommon and usually occurred after IA. These included respiratory distress (*n* = 5), croup-like cough (*n* = 6), and stridor (n = 6). The majority of these were managed conservatively with observation, with or without medical therapy including systemic steroids and inhaled medications. One patient required intubation, repeat endoscopy and drainage of a seroma following IA.

## Discussion

The available literature concerning LC1 has grown considerably in recent years, with several authors advocating various treatment algorithms and therapeutic modalities. Our results will help to clarify the role of IA and ER via a proposed management algorithm that can be further validated prospectively.

Overall the majority of patients had significant symptom improvement when managed by our algorithm, with two thirds having complete symptoms resolution. This success rate is similar or slightly higher than similar studies that made use of both IA and ER [9, 12]. In terms of non-responders, we found that the only independent predictor of treatment failure was tube feeding prior to initial evaluation. This is in contrast to Thottam et al. who found that silent aspirators were more likely to fail treatment, however that group did not specifically examine tube feeding as a predictor [12]. Although there was an association between silent aspiration and treatment failure on univariate analysis in our study, it was not an independent predictor of failure in the multivariate analysis.

One of the controversies surrounding LC1 is the diagnosis itself. Although classically described as an anatomic defect in the posterior laryngeal musculature [1], there is debate within the literature as to the role of an “anatomic” versus a “functional” cleft. This is in recognition of the fact that not all patients with a deep inter-arytenoid notch have signs and symptoms of aspiration or SD [9]. Additionally, some authors have argued that augmentation of the posterior larynx can improve clinical aspiration and SD even without definitive evidence of an anatomic cleft [16], as well as in the setting of a ‘deep inter-arytenoid notch.’ [17] One recent study attempted to measure the mucosal height of the posterior inter-arytenoid space and correlate this with the need for thickened feeds however no significant association was found [18]. In our experience, a blend of anatomic and functional cleft approaches serves the clinician best. In the present study, patients were considered for IA or ER if there was definitive evidence of SD without another identifiable cause, and a deep inter-arytenoid notch or “functional cleft” as well as a clear anatomic LC1, as other authors have reported [16]. However, the threshold for the diagnosis and treatment of LC1 remains an area in need of further clarification in the literature.

The concept of a diagnostic IA to determine the significance of a LC1 and predict response to ER has also gained attention in recent studies [3, 9, 10]. These authors argued that a favourable response to IA, supports the clinical significance of their LC1 and their candidacy for an ER. Indeed, we also identified that patients who have a transient improvement with IA do tend to do well with ER (Fig. 4). However, in contrast to the other studies, the majority of patients who failed to respond to IA in our study still had a favourable response to ER; thus, a failure to respond to IA should not necessarily preclude ER.

Another area where there is a lack of clarity is the type and duration of conservative measures that are used prior to endoscopic intervention. Some authors have advocated for SLB for all patients with SD, whereas others use it only for patients with posterior aspiration, and still others try conservative methods prior to any airway endoscopy [3, 6, 9, 19]. We believe that the strength of approach is that it is pragmatic, as it takes into account the presence of red flags which warrant more urgent endoscopy, as well as the route of feeding at initial evaluation. We propose that patients who are tube fed warrant earlier intervention with definitive repair, in order to rehabilitate the swallowing as early as possible. For patients that do not meet criteria for early intervention, we advocate a three months trial of conservative measures, as this will allow enough time to properly elucidate the response to treatment and save a significant number of endoscopic procedures.

Another key advantage of the algorithm presented here is a single stage diagnostic and therapeutic endoscopy. Other algorithms have used a diagnostic SLB to confirm the presence of LC1 followed by conservative measures and another airway endoscopy for treatment of the cleft if warranted [3, 6]. Although we acknowledge that a single stage procedure requires careful, flexible planning and an involved consent process, it also has clear advantages of limiting the number of anaesthetics and providing symptom relief earlier [20].

Another important feature of this study was the relative homogeneity of the patient population, as other studies have included a heterogeneous mix of patients with several severe comorbidities that can obscure the management of the LC1 [10]. Of particular note is the high prevalence of associated neurological conditions reported in other studies, such as as Thottam et al. (27%) [12] and Kennedy et al. (60%) [10], compared with our own cohort of 8%.

The fact that patients who were tube fed were less likely to respond to treatment is not surprising, as those patients tend to be more complex and have a de-conditioned swallowing mechanism from the use of an alternate route. In addition, the presence of the tube itself (nasogastric or nasojejunal) may itself alter swallowing mechanics. However, we cannot relate the presence of a tube preoperatively to a particular clinical threshold, as the Otolaryngology team did not necessarily participate in the decision to insert the tube in all cases. Thottam et al. identified that silent aspirators were more likely to fail treatment with IA which was not reproduced here [12]; however comparisons with our cohort are limited because it included patients that had ER in addition to IA.

This study does have weaknesses, including the fact that it was a single centre and single surgeon experience, with a limited sample size. This may have played a role in the lack of significant association between some pre-operative variables and treatment failure. The retrospective design also limits the ability of the algorithm to be properly validated, which should be done in a follow-up prospective study as we intend. However, we demonstrated steps that aimed at addressing reduction of general anaesthesia, combining multi-disciplinary consults and procedures and choice of interventions that respond to clinical severity. The controversy that exists around the diagnosis of LC1 and methods of its evaluation can limit the generalizability to other centres who use different methods or have different criteria for establishing the diagnosis. There is also disagreement on the appropriate time period for “conservative management,” and some other centres may argue for a longer treatment duration.

The results of the regression analysis should be interpreted with caution, as various factors that are markers for disease severity can interact with one another and skew the results. Various factors that were tested were different from other studies and indeed other papers never stated clearly all they included in their models. We also did not have consistent post-operative VFSS or FEES data with which to compare pre-operative findings with, for the reasons noted in the methods section. Indeed one author did report on an algorithm designed to reduce the number of VFSS performed [21]. The results from the patient cohort that did have both pre and post-operative swallowing data was favourable with the majority of patients having improvement in instrumental evaluation findings; however it should be noted that due the pragmatic nature of the decision to perform post-operative FEES, these patients did tend to have more abnormal pre-operative evaluations and/or more severe symptoms prior to management of the LC1 [17].

## Conclusion

LC1 can be effectively managed with a combination of IA and ER with very good results in properly selected patients. A single step diagnostic and therapeutic airway endoscopy is a pragmatic and useful practice that reduces the number of anesthetics and procedures. Failure to respond to IA does not appear to preclude ER, and both have their role in the management of LC1. Patients who are tube fed prior to evaluation seem to have a higher probability of treatment failure. The management algorithm presented here achieved favourable results and lends itself to further prospective evaluation.

## Data Availability

Data available from corresponding author upon request.
